# Deciphering the heterogeneity and plasticity of the tumor microenvironment in liver cancer provides insights for prognosis

**DOI:** 10.3389/fphar.2025.1495280

**Published:** 2025-01-30

**Authors:** Yihao Sun, Guojuan Shi, Jian Yang, Chun-Zhong Zhou, Chuhan Peng, Yu-Hong Luo, Ying Pan, Rui-Qi Wang

**Affiliations:** ^1^ Department of Pharmacy, Zhuhai People’s Hospital (The Affiliated Hospital of Beijing Institute of Technology, Zhuhai Clinical Medical College of Jinan University), Zhuhai, China; ^2^ Guangdong Provincial Key Laboratory of Tumor Interventional Diagnosis and Treatment, Zhuhai Institute of Translational Medicine, Zhuhai People’s Hospital (The Affiliated Hospital of Beijing Institute of Technology, Zhuhai Clinical Medical College of Jinan University), Zhuhai, China; ^3^ Department of Nephrology, Zhuhai People’s Hospital (The Affiliated Hospital of Beijing Institute of Technology, Zhuhai Clinical Medical College of Jinan University), Zhuhai, China; ^4^ Department of Respiratory and Critical Care Medicine, Zhuhai People’s Hospital (The Affiliated Hospital of Beijing Institute of Technology, Zhuhai Clinical Medical College of Jinan University), Zhuhai, China; ^5^ Canyon Crest Academy, San Diego, CA, United States; ^6^ Department of Oncology, Zhuhai People’s Hospital (The Affiliated Hospital of Beijing Institute of Technology, Zhuhai Clinical Medical College of Jinan University), Zhuhai, China

**Keywords:** liver cancer, single-cell, cancer-associated fibroblasts, metastasis, prognosis

## Abstract

Liver cancer exhibits diverse molecular characteristics and distinct immune cell infiltration patterns, which significantly influence patient outcomes. In this study, we thoroughly examined the liver cancer tumor environment by analyzing data from 419,866 individual cells across nine datasets involving 99 patients. By categorizing patients into different groups based on their immune cell profiles, including immune deficiency, B cells-enriched, T cells-enriched and macrophages-enriched, we better understood how these cells change in various patient subgroups. Our investigation of liver metastases from intestinal cancer uncovered a group of mast cells that might promote metastasis through pathways like inositol phosphate metabolism. Using genomic and clinical data from The Cancer Genome Atlas, we identified specific cell components linked to tumor characteristics and genetics. Our detailed study of cancer-associated fibroblasts (CAFs) revealed how they adapt and acquire new functions in the tissue environment, highlighting their flexibility. Additionally, we found a significant connection between CAF-related genes and the prognosis of hepatocellular carcinoma patients. This research provides valuable insights into the makeup of the liver cancer tumor environment and its profound impact on patient outcomes, offering fresh perspectives for managing this challenging disease.

## 1 Introduction

Liver cancer represents a formidable challenge in oncology, with an estimated 906,000 new cases and nearly 830,000 deaths each year (Rumgay et al., 2022). The disease is characterized by extensive molecular heterogeneity between individuals and within the same tumor in a single patient (Losic et al., 2020). This intricate variability renders the treatment of liver cancer a complex undertaking and impairs our understanding of its origins and progression. To effectively address this challenge, it is imperative to comprehensively explore the heterogeneous nature of liver cancer. Such an investigation has the potential to unveil the underlying mechanisms driving liver cancer development. It may pave new avenues for innovative therapeutic strategies in the fight against this aggressive malignancy.

The heterogeneity of the tumor immune microenvironment in liver cancer has been a hot topic in cancer research. In previous studies, the heterogeneity of the tumor immune microenvironment was mainly characterized based on pathological analysis and bulk transcriptome sequencing (Gao et al., 2021; Dong et al., 2022; Gao et al., 2019), which cannot accurately resolve immune cell composition and may ignore the critical subsets present at lower frequencies. With the development of single-cell sequencing technology, the heterogeneity of the liver cancer immune microenvironment has been further appreciated in recent years. A recent single-cell sequencing technology study conducted a detailed analysis of the liver cancer immune microenvironment and identified four subtypes: immune-suppressive, immune-active, mixed, and normal (Xue et al., 2022). Additionally, it has been suggested that the heterogeneity of the liver cancer immune microenvironment may be an essential factor contributing to drug resistance (Maacha et al., 2019), recurrence (Zhang et al., 2018), and poor prognosis (Liu et al., 2020). Overall, the heterogeneity of the tumor immune microenvironment in liver cancer is a complex and vital field that requires further investigation and exploration.

Cancer-associated fibroblasts (CAFs) are a distinct group of fibroblasts that reside within the tumor microenvironment and promote cancer progression through the secretion of various cytokines and signaling molecules (Sahai et al., 2020; Chen et al., 2021). In liver cancer, the abundance and activity of CAFs are closely linked to patient prognosis (Affo et al., 2017). It has been shown that CAFs can facilitate liver cancer development through multiple mechanisms, including secretion of growth factors, inhibition of immune response, promotion of cell proliferation, and angiogenesis (Kubo et al., 2016; Yin et al., 2019). In addition to promoting liver cancer development, CAFs have also been implicated in drug resistance (Chen and Song, 2019), a significant obstacle in liver cancer therapy. It has been demonstrated that CAFs can mediate drug resistance of liver cancer cells through the secretion of various resistance-related molecules (Uchihara et al., 2020; Yu et al., 2017).

Furthermore, CAFs interact with immune cells to influence liver cancer immune response and the efficacy of immunotherapy (Liu et al., 2019). Current research on the function of CAFs in liver cancer has mainly focused on cytokines and signaling pathways. However, the underlying molecular mechanisms by which CAFs promote liver cancer development remain elusive and require further investigation. Additionally, developing more effective therapeutic strategies targeting CAFs to inhibit liver cancer growth and drug resistance remains critical for future research.

This study used data from 419,866 individual cells across nine datasets to examine the liver cancer tumor environment. The results show that liver cancer has diverse molecular characteristics and distinct immune cell infiltration patterns that can significantly impact patient outcomes. The immune cell profiles of patients were classified into different groups, including immune deficiency, B cells-enriched, T cells-enriched and macrophages-enriched, which helped researchers gain a deeper understanding of how these cells change in various patient subgroups. We also identified specific cell components linked to tumor characteristics and genetics using genomic and clinical data from TCGA. Additionally, the study provided valuable insights into the makeup of the liver cancer tumor environment and its impact on patient outcomes, offering fresh perspectives for managing this challenging disease.

## 2 Materials and methods

### 2.1 Datasets collection

The Log2-normalized expression matrix for liver cancer in The Cancer Genome Atlas (TCGA), along with relevant clinical and phenotype data, was obtained from the official UCSC Xena website (https://xenabrowser.net/datapages/). Additionally, transcriptomic data, in conjunction with clinical records, originating from 159 paired tumor and normal tissue samples obtained from Chinese patients (CHCC) afflicted with Hepatitis B Virus (HBV)-associated Hepatocellular Carcinoma (HCC), were procured through the National Omics Data Encyclopedia database (https://www.biosino.org/node/) under accession number OEP000321 [20]. Furthermore, we accessed the LIRI-JP dataset (ICGC), encompassing transcriptome profiles from 231 HCC specimens, via the International Cancer Genome Consortium Data Portal (https://dcc.icgc.org/). In addition, we acquired gene expression microarray data and comprehensive clinical annotations of GSE14520, which comprises 221 HCC samples, from the Gene Expression Omnibus repository (https://www.ncbi.nlm.nih.gov/geo/). For single-cell analyses, we collected liver cancer datasets from GEO, including GSE125449 (comprising eight samples), GSE140228 (comprising five samples), GSE112271 (comprising two samples), GSE178318 (comprising six samples), GSE151530 (comprising 23 samples), GSE166635 (comprising two samples) and GSE164522 (comprising ten samples). Furthermore, we obtained single-cell data from liver cancer samples (20 samples) through the Single-Cell Colorectal Cancer Liver Metastases (scCRLM) Atlas website (http://www.cancerdiversity.asia/scCRLM/) and one single-cell data from colorectal cancer samples GSE132465 (23 samples).

### 2.2 Preprocessing of single-cell datasets

Individual sample quality control was meticulously executed, eliminating substandard data instances, such as those originating from damaged or diseased cells, vacant droplets devoid of captured cells, and doublets where multiple cells were concurrently captured. Typically, inferior cells or empty droplets exhibit a paucity of expressed genes, whereas doublets are prone to manifest an increased gene count. Additionally, inferior or diseased cells often exhibit elevated mitochondrial gene expression. Quality filtering was conducted based on two criteria: (1) the stipulation that cells must express a minimum of 300 genes and (2) the prerequisite for a mitochondrial-to-ribosomal gene ratio of less than 20%. Subsequently, data underwent normalization, dimensionality reduction, and clustering procedures.

Given that single-cell sequencing mandates that each barcode label uniquely corresponds to a single viable cell, occasional scenarios arise where two or more cells share a barcode, identified as doublets. To address this, DoubletFinder (McGinnis et al., 2019) was employed for the identification and subsequent removal of potential doublets. Ultimately, our analysis retained 200,466 single cells from 72 distinct patients and spanning six distinct datasets.

### 2.3 Odds ratios (OR) calculation

We employed the odds ratios (ORs) method as Zheng et al. (2021) detailed to evaluate meta-clusters’ proclivities in terms of tissue distribution. To evaluate the distribution of specific cell types across different tissues, we assessed the likelihood of a particular cell type being enriched or depleted in a given tissue. This analysis involved creating a table that compared the number of cells of a specific type in the tissue of interest to their numbers in other tissues, while also considering the distribution of all other cell types in those tissues. For each cell type and tissue combination, we calculated an OR to quantify the association between the cell type and the tissue. The OR measures whether a specific cell type is more likely to appear in one tissue compared to others. To ensure the statistical robustness of the results, we used Fisher’s exact test to evaluate the significance of the association and adjusted the resulting p-values for multiple comparisons using the false discovery rate (FDR) method. An OR greater than 1.5 indicates that the cell type is more likely to be found in the tissue of interest, signifying enrichment. An OR less than 0.5 suggests that the cell type is less likely to be present in that tissue, indicating depletion or exclusion.

### 2.4 Tumor microenvironment-based subtype

Using the integrated single-cell expression matrix derived from the amalgamation of six datasets, we proceeded with cell subpopulation annotation and the computation of cell subpopulation proportions in each sample. Subsequently, we conducted sample clustering alongside their respective matrices of cell subpopulation proportions utilizing the hclust function. This enabled us to categorize the samples into distinct subtypes, including immune-deserted, B cell-enriched, T cell-enriched, and macrophage-enriched subtypes, based on the prevalence of each cell type within the individual clusters.

### 2.5 Pathway activity estimation

Utilizing the integrated single-cell expression matrix derived from the combination of six datasets, we isolated the expression matrix for subsequent analysis from the Seurat object. Cancer hallmark pathways and Kyoto Encyclopedia of Genes and Genomes (KEGG) pathways and their associated gene information were obtained from the Gene Set Enrichment Analysis (GSEA) database (Subramanian et al., 2005). Employing the R package GSVA (Hänzelmann et al., 2013), we used the expression matrix to conduct a single-sample Gene Set Enrichment Analysis (ssGSEA) on each sample. The pathway activity level for each sample was determined by considering the enrichment score of each pathway.

### 2.6 Transcription factor activity score

Utilizing the integrated single-cell expression matrix from the amalgamation of six datasets, we isolated the expression matrix from the Seurat object for subsequent analysis. Regulatory associations between transcription factors and their targets and the corresponding gene information were acquired from the GSEA database. Employing the R package GSVA, we conducted ssGSEA for each sample, utilizing the expression matrix. The transcription factor activity level for each sample was determined by considering the enrichment score of each transcription factor.

### 2.7 Scissor analysis

The Scissor algorithm (Sun et al., 2022) introduces an innovative methodology for analysing single-cell data, capitalizing on an extensive array of phenotypic data to discern highly phenotypically correlated cell subpopulations within single-cell sequencing datasets. Significantly, Scissor identifies cells linked to specific phenotypes, displaying distinct molecular profiles characterized by key marker genes and pertinent biological processes associated with the respective phenotype. Notably, the Scissor algorithm eliminates the necessity for unsupervised clustering in single-cell data analysis, thereby mitigating subjectivity in determining cluster numbers and resolution. Moreover, Scissor provides a versatile framework for seamlessly integrating diverse external phenotypic data into the analysis pipeline, facilitating hypothesis-free identification of cell subpopulations with clinical and biological relevance. In this investigation, we harnessed mutation, survival, and gene expression data from the TCGA database for liver cancer to predict the cell subpopulations most closely linked to specific mutations, overall survival, and progression-free survival, with all parameters employed at their default settings.

### 2.8 CAF signature-based risk score

Using the R function FindAllMarkers, we computed differentially expressed genes (DEGs) distinguishing CAFs from other cell types. The top 40 genes exhibiting the highest log2 (FoldChange) were selected for constructing the risk model. Employing the R package “glmnet v4.1-2″, we conducted LASSO-Cox regression analysis on the expression of these 40 genes in TCGA LIHC samples to identify candidate genes. Subsequently, after 1,000 rounds of adjustment and cross-validation, a 9-gene signature was established. A linear combination of the expression of these characteristic genes was employed to compute the risk score for each patient, with the minimum criterion determining the regression coefficient. The risk score formula is defined as follows: risk score = k1 * x1 + k2 * x2 + . + ki * xi (i = n), where i denotes each selected gene, k signifies the regression coefficient, and x represents the expression level. Specifically, this study defines the final model as risk score = 0.091*THY1 + 0.199*CNN3 + 0.089*IGFBP3-0.066*IGFBP7-0.031*SERPING1-0.066*C7-0.017*RARRES2-0.096*C1S-0.015*CXCL14. Furthermore, leveraging the optimal survival cutoff derived from TCGA data, we stratified the samples into high-risk and low-risk groups.

### 2.9 Bioinformatics analysis

Assessment of epithelial cell copy number variation was conducted using the infervcnv package. Evaluation of the mutation landscape in TCGA liver cancer samples was performed utilizing the R package maftools (Mayakonda et al., 2018). Pseudo-time series analysis was executed using the R package monocle3 (Cao et al., 2019). Survival analysis and Kaplan-Meier curve plotting were carried out using the R package survival (Therneau and Lumley, 2015). Time-dependent receiver operating characteristic (ROC) curve analysis was conducted using the R package timeROC. The generation of nomograms and calibration curves was achieved using the R package rms.

## 3 Results

### 3.1 Establishment of a combined single-cell atlas for liver cancer

**FIGURE 1:**
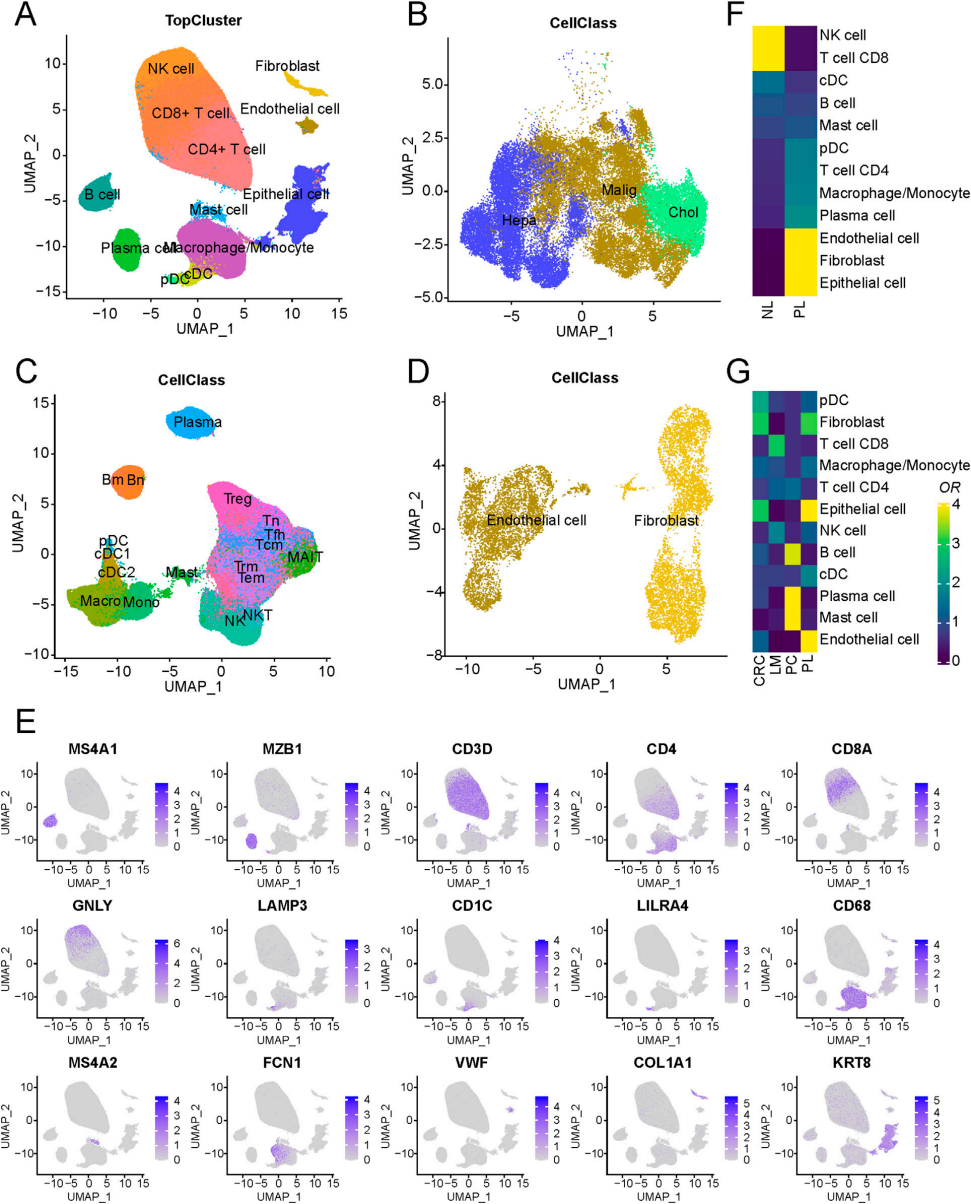
Schematic outline of the overall concept used in this study. **(A)** Overview of the core liver cancer atlas depicted as uniform manifold approximation and projection (UMAP) plots. **(B)** Overview of the epithelial components depicted as UMAP plots. **(C)** Overview of the immune components depicted as UMAP plots. **(D)** Overview of the stromal components depicted as UMAP plots. **(E)** UMAP of cell-type marker genes used for cell-type annotation. **(F)** Cell-type composition differences between tumor and normal-adjacent tissues. **(G)** An analysis of cell-type of LM, PL, PC, and CRC.

In this study, publicly accessible single-cell sequencing datasets for liver cancer were curated, and stringent quality control measures, including the removal of doublets, were applied, resulting in the inclusion of 419,866 single cells from 99 patients spanning nine datasets, establishing a fundamental single-cell atlas for liver cancer. The harmony algorithm (Korsunsky et al., 2019) was employed to effectively mitigate batch effects, ensuring the robustness of the integrated dataset with minimal batch influence, as visually demonstrated in Supplementary Figure S1A. Utilizing specific marker genes for annotation, the cells were categorized into three major groups: epithelial, immune, and stromal cells (as presented in Figures 1A–D). These included epithelial cells, endothelial cells, fibroblasts, B cells, plasma cells, mast cells, macrophages/monocytes, dendritic cells, natural killer cells, and T cells. Notably, the markers distinguishing these cell populations were identified, such as KRT8 for epithelial cells, VWF for endothelial cells, COL1A1 for cancer-associated fibroblasts, MS4A1 for B cells, MZB1 for plasma cells, MS4A2 for mast cells, CD68 for macrophages/monocytes, LAMP3 for dendritic cells, CD3D for T cells, and GNLY for natural killer cells. Cells displaying high expression of genes associated with cell proliferation, such as MKI67 and TOP2A, were intentionally excluded to ensure the robustness of the dataset.

Regarding cell composition, T cells were found to comprise the majority of cells, followed by epithelial and B cells, with their proportions illustrated in Supplementary Figure S1B. Nonetheless, substantial variability in immune and epithelial cell proportions was observed among different samples within the datasets (Supplementary Figure S1C; Supplementary Table S1). A comparison of tumour and normal-adjacent tissue samples revealed distinct characteristics in tumor samples, marked by elevated proportions of endothelial cells and fibroblasts alongside decreased NK and CD8^+^ T cell proportions (Figure 1F). These findings suggest a potential immunosuppressive microenvironment in liver cancer, contributing to its malignant progression (Oura et al., 2021). Additionally, an examination of cellular compositions in metastatic lesions of livers (LM), primary liver (PL), primary colorectal cancers (PC) and non-metastatic colorectal cancers (CRC) unveiled a significant increase in mast cell content in PC and endothelial cell content in PL (Figure 1G). Such differences between the microenvironments of metastatic and primary lesions may arise from adverse conditions, including regulating immune response and angiogenesis in the tumor microenvironment (Li X. et al., 2021), promoting tumor cell adhesion, vascular penetration, and migration to distant tissues. Further stratification of epithelial cells based on tissue origin and copy number variation (CNV) led to their classification into two distinct groups: normal and malignant epithelial cells. Furthermore, an evaluation of gene expression related to normal liver marker ALB demonstrated markedly heightened expression of ALB in normal epithelial cells, indicating that the tumor tissue has lost its normal function.

### 3.2 Deciphering unique immune phenotypes through single-cell analysis

**FIGURE 2:**
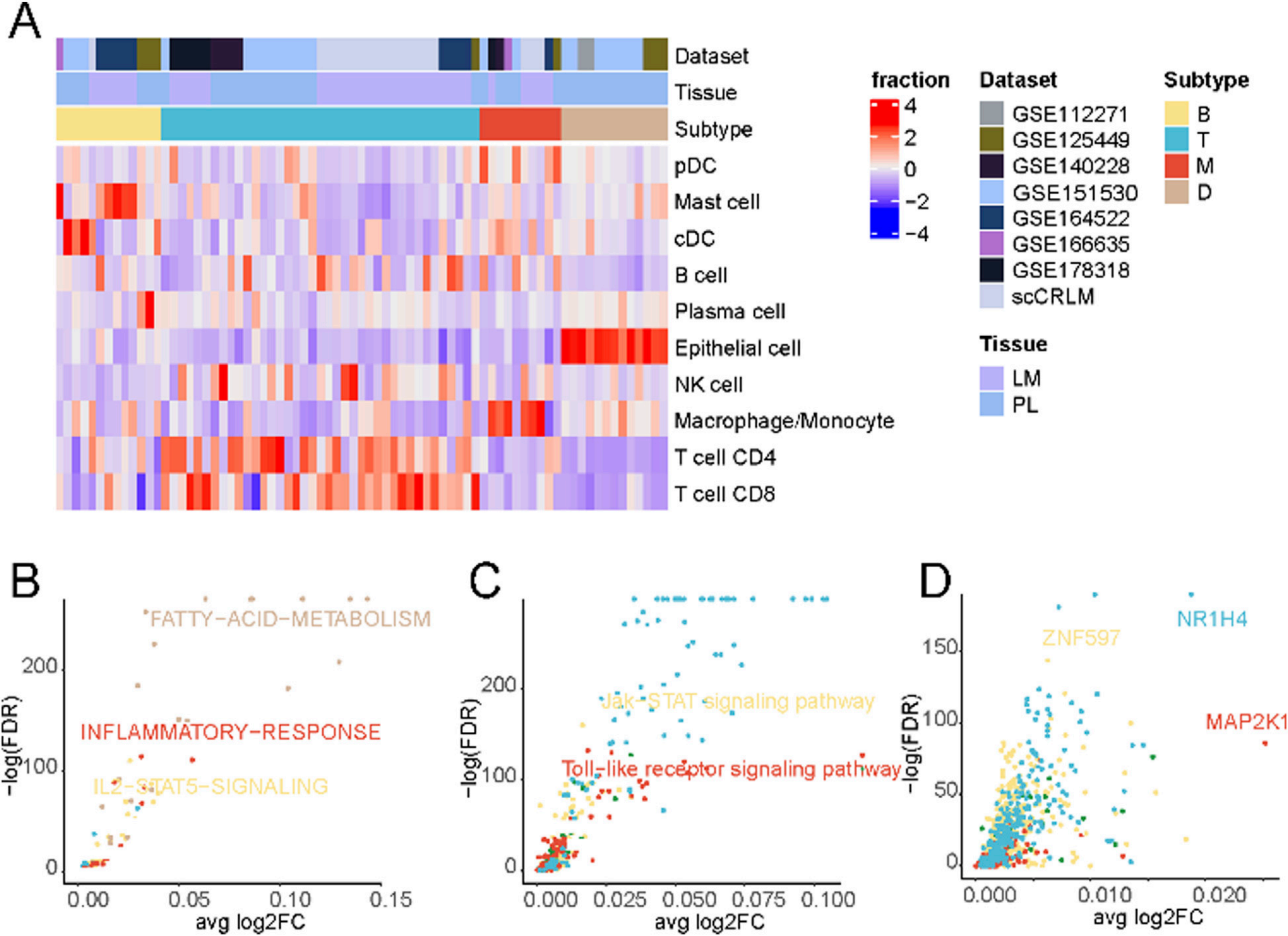
Tumor immune phenotypes in liver cancer. **(A)** Patient characteristics and stratification of the tumor immune phenotypes. The original datasets provide tumor origins. **(B)** Differential activation of cancer hallmark pathways between the four tumor immune phenotypes in cancer cells. **(C)** Differential activation of KEGG pathways in cancer cells between the four tumor immune phenotypes in cancer cells. **(D)** Differential activation of transcription factors in cancer cells between the four tumor immune phenotypes in cancer cells.

Subsequently, patient stratification was undertaken based on each sample’s distinctive immune cell composition. As delineated in Figure 2A, a four-fold categorization of patients emerged, encompassing immune-deserted, T cell-infiltrated, B cell-infiltrated, and macrophage-infiltrated types, each defined by variations in the prevalence of enriched cell types. The immune-deserted type (D-type) primarily comprised epithelial cells, with other immune cell populations maintained at relatively lower levels. In contrast, the macrophage-infiltrated type (M-type) exhibited a pronounced increase in macrophages/monocytes. The T cell-infiltrated type (T-type) showcased the highest levels of T cell infiltration, encompassing CD4^+^ and CD8^+^ T cells. Lastly, Both cell-infiltrated types (B-type) displayed a relatively heightened proportion of mast and cDC cells (Supplementary Figure S2).

A comparison of differentially enriched pathways among tumor cells across subtypes was performed to elucidate the distinctive tumour cell gene expression profiles in each subtype. As shown in Figure 2B and Supplementary Table S2, tumor cells in D-type expressed significantly higher levels of fatty acid metabolism signaling pathways. Tumor cells often express higher levels of active kinases that can promote the synthesis and accumulation of fatty acids (Zhao et al., 2020; Li W. et al., 2021), thereby suppressing immune responses (Wu et al., 2021; Zhao et al., 2021). Additionally, tumor cells can regulate key molecules in fatty acid metabolisms, such as fatty acid synthase and lipoxygenase, to affect the activation and function of immune cells, thus creating an immunosuppressive microenvironment (Hu et al., 2020). Tumor cells in M-type displayed a high inflammatory response. Macrophages, as one of the key cells in the inflammatory response, can secrete a variety of biologically active mediators, such as cytokines, chemokines, and growth factors, which can promote inflammation, attract and activate other immune cells, thereby initiating and amplifying the inflammatory response (Korbecki and Bajdak-Rusinek, 2019).

Contrasting the distinctive cellular metabolic pathways (Figure 2C; Supplementary Table S3), The Toll-like receptor (TLR) signaling pathway was significantly upregulated in the M-type. When macrophages recognize pathogen-associated molecular patterns through surface-expressed TLR receptors, a series of signal transduction pathways are triggered, initiating and amplifying inflammatory responses (Lauterbach et al., 2019). These findings suggest that macrophages and the TLR signaling pathway play crucial roles in initiating and regulating inflammation, and their interactions and regulations may have important implications for immune system function and disease development.

Finally, an evaluation of differentially enriched transcription factors (Figure 2D; Supplementary Table S4) revealed notable enrichment of NR1H4 in the T cell-enriched subgroup. NR1H4 has been reported to be closely related to MYC expression and stability (Wang et al., 2022), and the activation of the MYC gene is crucial for T cells’ normal function and immune response (Saravia et al., 2020). Additionally, we observed significant activation of MAP2K1 in the M-type, which, according to previous literature, may regulate pulmonary macrophage inflammatory responses and resolution of acute lung injury (Long et al., 2019).

### 3.3 Crosstalk between tumor cells and TME

**FIGURE 3:**
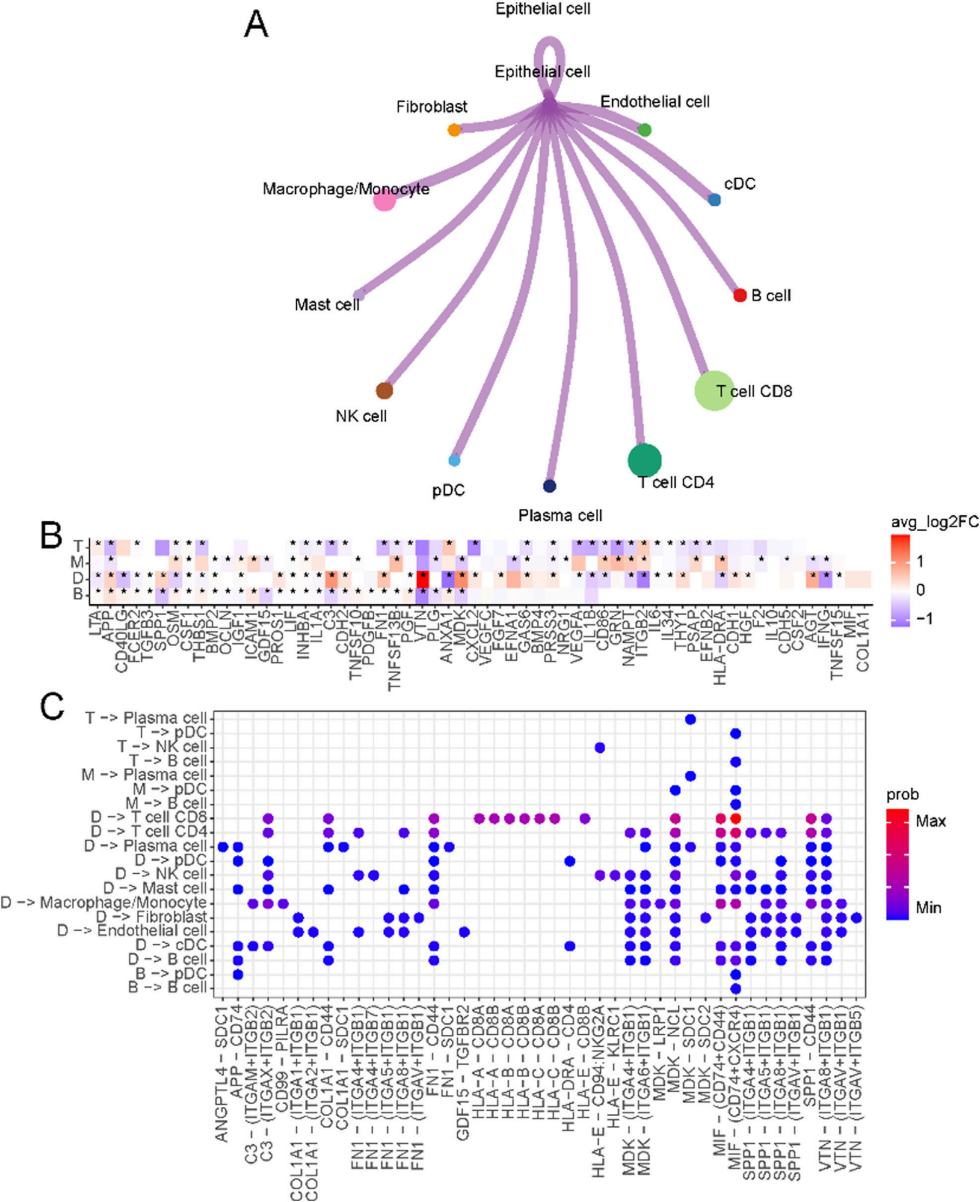
Crosstalk between epithelial cells and immune microenvironments. **(A)** Circos plot of the cellular crosstalk of cancer cells toward the major immune cells. **(B)** Differentially expressed ligands of cancer cells in each subtype (B, M, T, D) **(C)** Cancer-immune cell crosstalk in each patient subtype.

The crosstalk between tumor cells and immune cells in the microenvironment is the basis for studying the interaction between tumors and their surroundings. We first overviewed the connections between malignant epithelial cells and other cells in the immune microenvironment (Figure 3A; Supplementary Figure S3A). The specific ligand-receptor interactions are shown in Supplementary Figure S3B. In D-type tumors, the immunosuppressive microenvironment is shaped by the interaction of VTN (vitronectin) and MDK (midkine) (Figure 3B), with their respective receptors (ITGA8, ITGB1, and ITGB5) (Figure 3C). VTN-ITGB1 signaling plays a critical role in reducing immune cell activation and migration, creating an exclusion zone that hinders effective T cell infiltration (Palazzo et al., 2022). Similarly, MDK-LRP1 (Hu et al., 2024) and MDK-SDC1 (Tan et al., 2023) interactions promote pro-tumorigenic signaling and immune escape pathways, further reinforcing immune suppression in this subtype. The receptors of MDK include ITGB1, LRP1, NCL, SDC1, and SDC2. Most of these ligand-receptor interactions can only be found in D-type.

In T-type tumors, an elevated level of NAMPT (nicotinamide phosphoribosyltransferase) was observed. NAMPT emerges as a critical regulator of T cell metabolism and immune function. As a key enzyme in the NAD + biosynthesis pathway (Garten et al., 2015), NAMPT ensures the energy supply necessary for T cell proliferation and effector functions by maintaining intracellular NAD + levels. Beyond its metabolic role, NAMPT promotes the differentiation and activity of Th1 cells (Mercurio et al., 2021), which secrete pro-inflammatory cytokines such as interferon-gamma (IFN-γ) (Cope et al., 2011), thereby amplifying the cytotoxic activity of CD8^+^ T cells. This creates a tumor microenvironment that supports robust anti-tumor immunity. This dual role of NAMPT, both intracellularly and extracellularly, underscores its importance in establishing the enhanced immune response characteristic of T-type tumors.

To better understand the tumor microenvironment in liver cancer, we analyzed TME cell interactions in single-cell data, focusing on the four primary immune-related groups: B, D, M, and T. The interaction dynamics within the TME (Supplementary Figure S3D) showed that the T group exhibited strong T cell interactions, particularly involving CD8^+^ and CD4^+^ T cells, promoting an active immune response. In contrast, the M group showed predominant interactions between macrophages and fibroblasts, suggesting a tumor-promoting and immunosuppressive microenvironment. The B group and D group exhibited weaker or more limited interaction patterns, with the D group reflecting an immune-deserted environment. Using the marker genes of these groups, we classified TCGA liver cancer samples into these four groups based on their gene expression profiles and evaluated their clinical relevance. The survival analysis (Supplementary Figure S3E) revealed significant differences in overall survival among the four groups. Patients in the T group showed the best prognosis, likely due to the robust anti-tumor immune activity driven by T cell interactions. In contrast, the M group had the worst prognosis, likely due to the pro-tumorigenic roles of macrophages, which secrete factors that promote tumor growth, immune suppression, and extracellular matrix remodeling, creating a microenvironment that fosters tumor progression and metastasis. Patients in the B and D groups showed intermediate survival outcomes. These findings emphasize the importance of TME composition and interaction dynamics in shaping clinical outcomes and suggest that targeting macrophage-driven processes in the M group or enhancing T cell responses in other groups could improve patient outcomes in liver cancer.

### 3.4 Reshape of TME during liver metastasis of colorectal cancer

**FIGURE 4:**
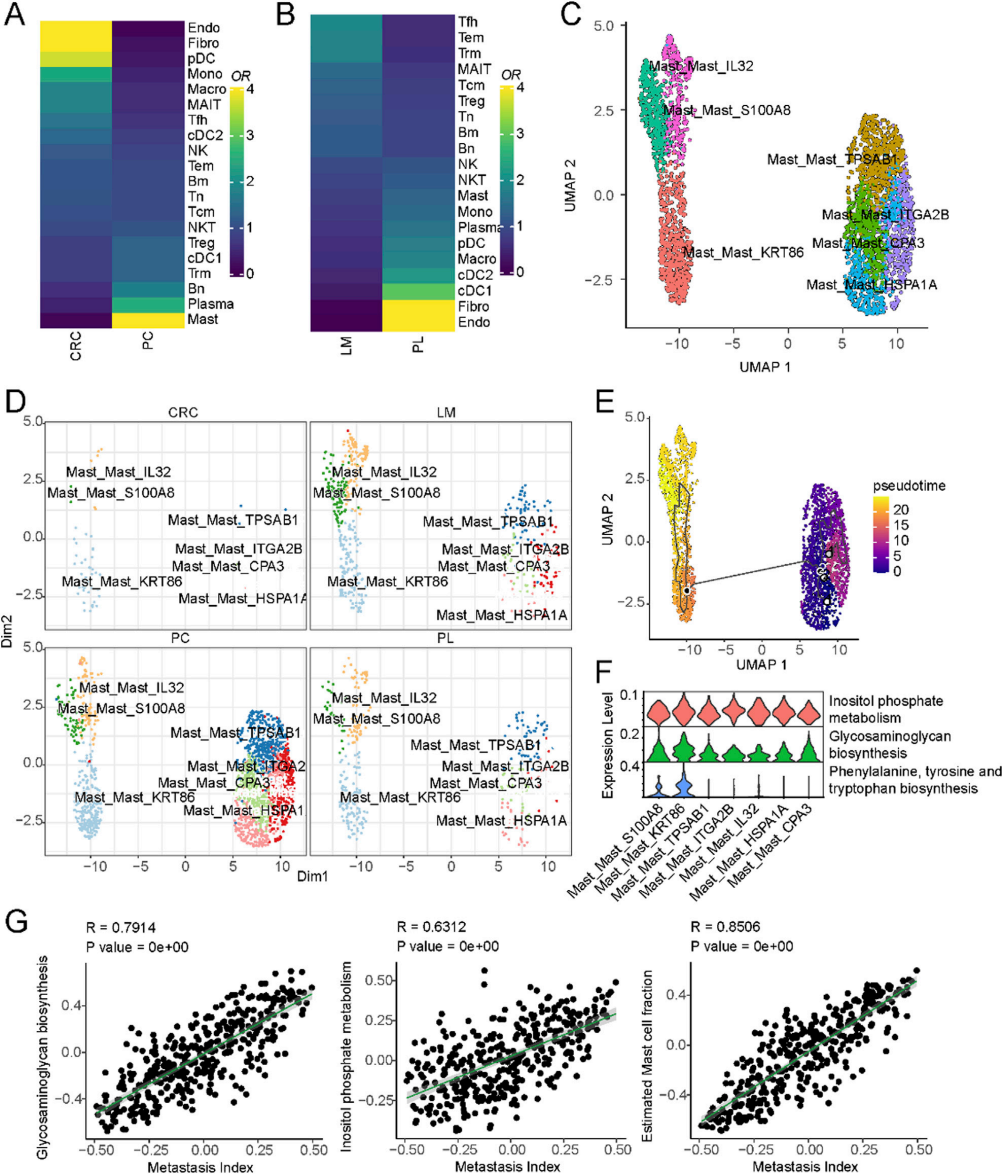
Mast cells and their association with liver metastasis in colorectal cancer. **(A)** Cell-type composition differences between non-metastatic and metastatic colorectal cancer. **(B)** Cell-type composition differences between primary liver and liver metastasis of colorectal cancer. **(C)** UMAP of all mast cells colored by different subclusters. **(D)** Comparison of cell fractions of mast cells among different tissue origins. **(E)** UMAP of all mast cells from the core liver cancer atlas with monocle vectors projected on top. **(F)** Differential activation of metabolic pathways in mast cells. **(G)** Correlation of metastatic index and metabolic pathway activities in colorectal cancer.

A subset of liver cancer arises from colorectal cancer metastasis to the liver, and the mechanism driving this metastasis involves many tumor microenvironmental factors. To explore the immune cell composition differences between the microenvironments of non-metastatic colorectal cancer, primary liver cancer, and colorectal cancer metastatic to the liver, we compared the immune cell populations in these three conditions. As shown in Figure 4A, mast cell populations were significantly enriched in colorectal cancer metastatic to the liver compared to non-metastatic colorectal cancer. A subset of mast cells was also present in liver metastatic lesions compared to primary liver cancer (Figure 4B). These results suggest mast cells may play a role in colorectal cancer metastasis to the liver. We further characterized the mast cells into two subsets based on differential marker expression (Supplementary Figure S4A). High IL32, S100A8, and KRT86 expression defined one subset. IL32 has been shown to induce pro-inflammatory cytokines and mediate chemotaxis of eosinophils and mast cells (Kempuraj et al., 2010), while S1008 has been reported to be associated with mast cell activation (Goyette and Geczy, 2011). Compared to the other subset, this population displayed a more activated state (Figure 4C). We then compared the composition of these mast cell subsets between non-metastatic colorectal cancer, primary liver cancer, and colorectal cancer metastatic to the liver. As shown in Figure 4D and Supplementary Figure S4B, this activated mast cell population was significantly expanded in colorectal cancer metastatic to the liver. Pseudo-time analysis suggested that these activated mast cells may represent a more differentiated state (Figure 4E).

Previous studies have reported that metabolic pathways are important in promoting colorectal cancer metastasis to the liver (La Vecchia and Sebastián, 2020). To investigate the potential role of metabolism in this process, we compared the activity of different metabolic pathways between these two subsets of cells. Inositol phosphate metabolism, Glycosaminoglycan biosynthesis, Phenylalanine, tyrosine and tryptophan biosynthesis were significantly upregulated in the activated mast cell population (Figure 4F). These pathways represent potential therapeutic targets to disrupt mast cell-mediated pro-metastatic effects. For example, inositol phosphate metabolism inhibitors, such as LiCl (lithium chloride), have shown promise in modulating cellular signaling and immune responses. Similarly, glycosaminoglycan biosynthesis inhibitors, including heparin analogs or beta-xylosides, could prevent mast cell degranulation and extracellular matrix remodeling, both critical for metastatic progression. We further validated these findings using primary colorectal cancer and liver metastatic lesions from different tissue sources (Supplementary Figure S4C). Finally, we interrogated the relationship between these metabolic pathways and colorectal cancer metastasis using The Cancer Genome Atlas (TCGA) colorectal cancer dataset. By obtaining pathway activity scores for each sample using single-sample gene set enrichment analysis (ssGSEA) and correlating these scores with metastasis-related gene signatures, we found that Inositol phosphate metabolism and Glycosaminoglycan biosynthesis were significantly correlated with colorectal cancer metastasis (Figure 4G). These results suggest that a subset of mast cells may promote colorectal cancer metastasis to the liver through metabolic pathways that regulate immune cell function.

### 3.5 Integration analysis reveals genotype-immune phenotype associations

In our subsequent analysis, we harnessed the innovative SCISSOR tool, which has recently emerged as a valuable resource for linking cell types, genetic profiles, and survival outcomes within the framework of single-cell sequencing data. Our primary objective was to unravel disparities in immune cell constituents across diverse phenotypic categories. Initially, we focused on the genetic mutation data and their interplay with cellular components. As illustrated in Supplementary Figure S5A, we compiled a catalogue of frequently occurring mutations in liver cancer based on TCGA mutation data, including noteworthy mutations such as TP53, CTNNB1, and APOB. Given the well-documented prevalence of TP53 and CTNNB1 mutations in liver cancer (Tornesello et al., 2013), we delved into their associations with immune cell composition (Figures 3A, B). Notably, TP53 mutations exhibited a significant positive correlation with mast and T cells while demonstrating a negative correlation with B cells and endothelial cells (Supplementary Figure S5B). These suggest that mutations in the TP53 gene can lead to the development of tumors, which can stimulate an immune response from mast cells and T cells, ultimately leading to the death of the tumor cells.

Additionally, TP53 can regulate the production of cytokines, thereby affecting the activation and function of mast cells and T cells (Hussain et al., 2007). On the contrary, CTNNB1 is the opposite, which is consistent with the mutual exclusivity of mutations in CTNNB1 and TP53 in many cases (Tornesello et al., 2013). Besides, APOB mutations exhibited a significant positive correlation with endothelial cells and fibroblasts while demonstrating a negative correlation with DC cells and macrophages (Supplementary Figure S5C).

**FIGURE 5:**
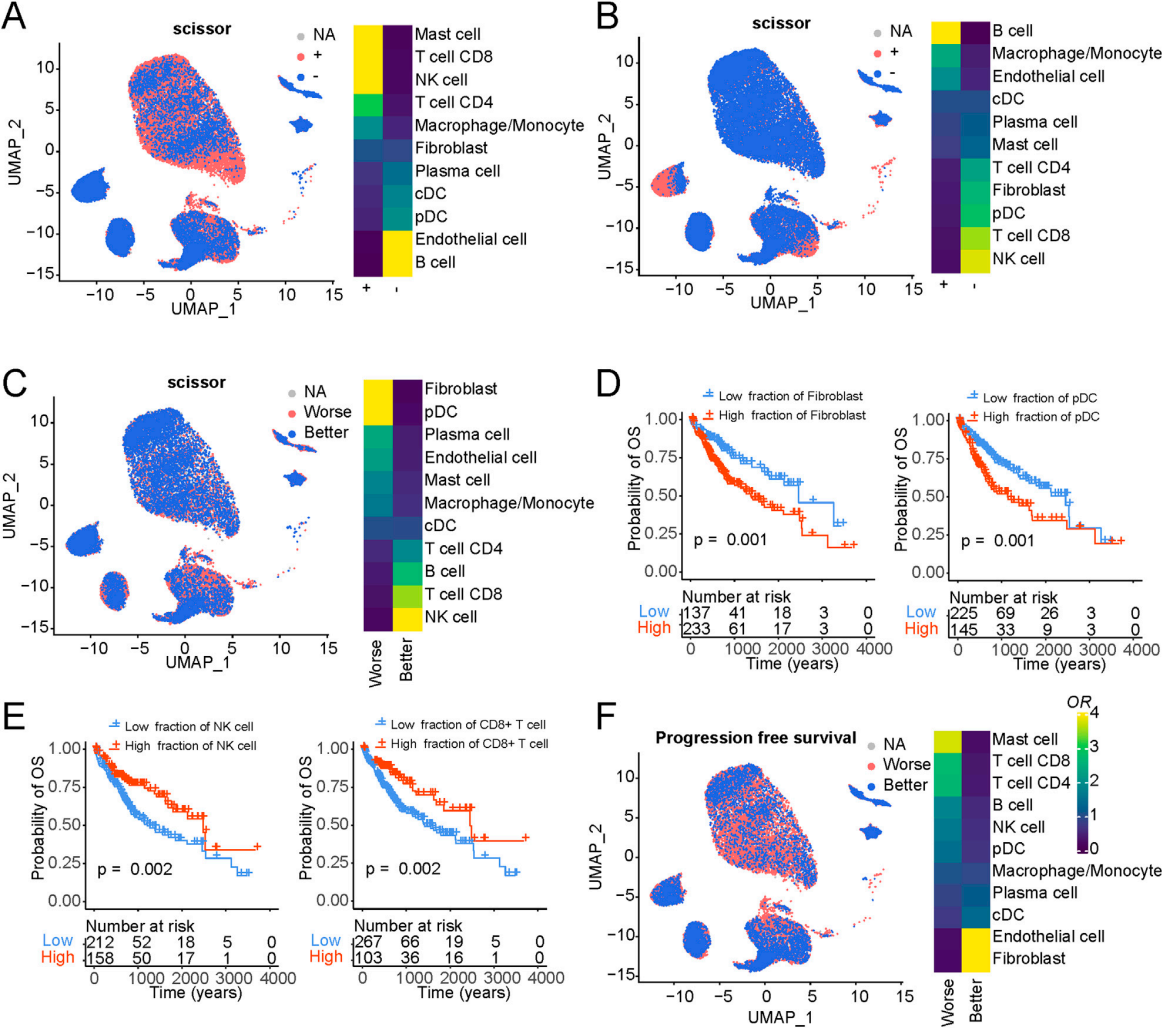
Association of cellular composition and distinct genotypes and survival in the TCGA data. **(A)** Association of cellular composition with TP53 mutation in patients with LIHC. **(B)** Association of cellular composition with CTNNB1 mutation in patients with LIHC. **(C)** Association of cellular composition with overall survival. **(D)** Kaplan-Meyer plot of patients with high and low Fibroblasts/pDC cell fractions of TCGA patients with liver cancer as determined by deconvolution with xCell. P-value has been determined using CoxPH regression using tumor stage and age as covariates. **(E)** Kaplan-Meyer plot of patients with high and low NK/CD8+ T cell fractions of TCGA patients with liver cancer as determined by deconvolution with xCell. P-value has been determined using CoxPH regression using tumor stage and age as covariates. **(F)** Association of cellular composition with overall survival.

Expanding our inquiry to the linkage between immune cell components and overall survival outcomes (Figure 5C), we pinpointed specific cell populations significantly associated with adverse patient prognoses, notably fibroblasts and pDC. Conversely, NK and T cells emerged as robust indicators of improved patient survival. Our exploration extended to the estimation of distinct cellular components for each sample via xCell (Aran et al., 2017), leveraging TCGA liver cancer data, and we assessed their relationships with overall patient survival (Figures 5D, E and S5D). Our survival analysis was predicated on bulk data, and the phenotype associations rooted in single-cell data echoed consistent results. Among these, fibroblasts showcased the most robust association with unfavorable patient outcomes, underscoring the pivotal role of cancer-associated fibroblasts in instigating and advancing tumor development. Furthermore, as gauged by progression-free survival analysis, our phenotype associations substantiated similar trends (Figure 5F).

### 3.6 Plasticity and canonical functional properties of CAFs

**FIGURE 6:**
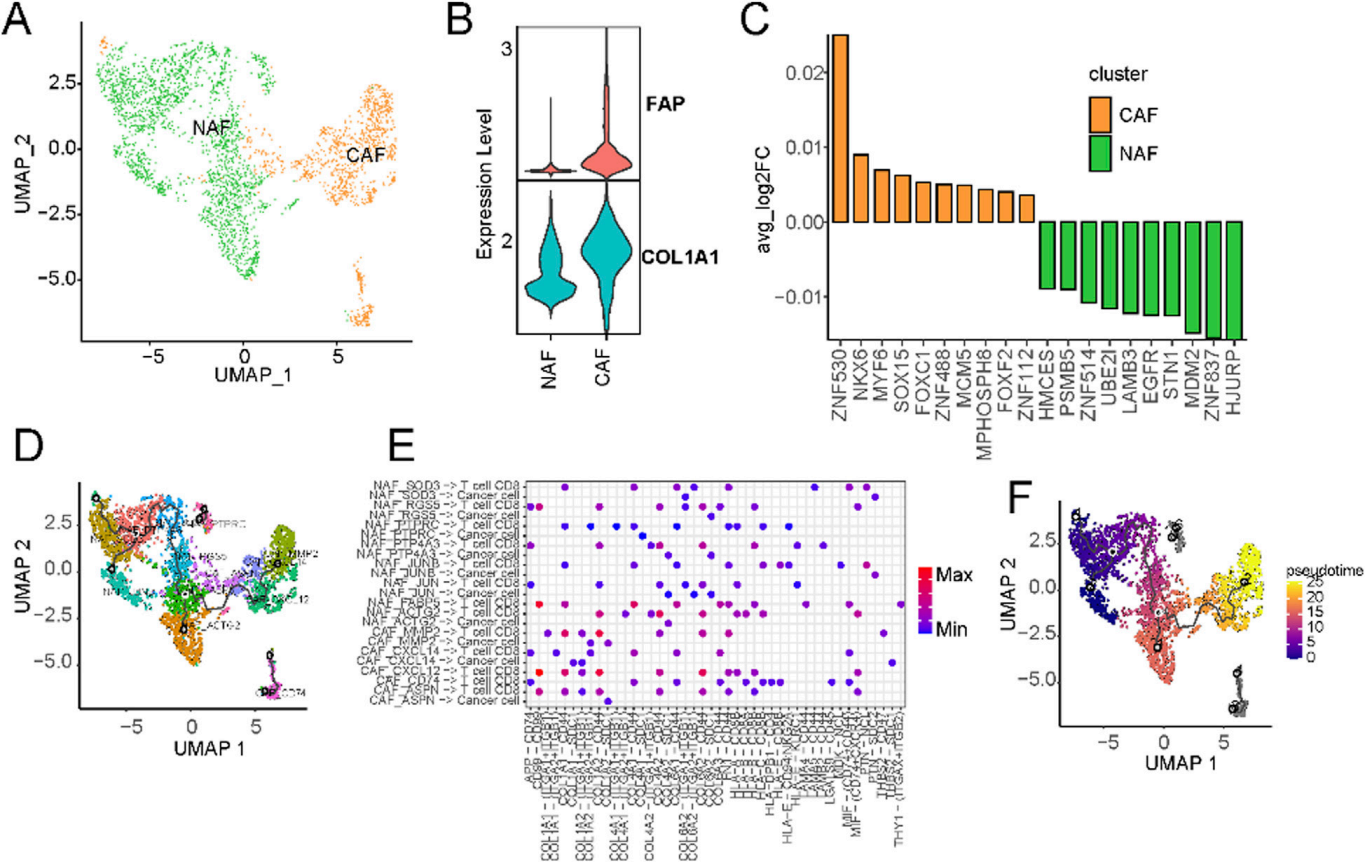
Characterization of cancer-associated fibroblasts using scRNA-seq. **(A)** UMAP of CAFs from the extended atlas was classified into cancer-associated fibroblasts (CAFs) and normal-associated fibroblasts (NAFs). **(B)** Expression levels of FAP and ACTA2 between CAFs and NAFs. **(C)** Transcription factor analysis of CAFs versus NAFs using ssGSEA. **(D)** UMAP of all fibroblasts colored by CAFs and NAFs subclusters. **(E)** Circos plot of the cellular crosstalk of fibroblast subclusters toward cancer cells and CD8^+^ T cells. **(F)** UMAP of all fibroblasts from the core liver cancer atlas with monocle vectors projected on top.

Given the robust association observed between fibroblast components and overall survival in liver cancer, we embarked on an exhaustive subcategorization of all fibroblasts, predicated on distinct marker expressions, culminating in the classification of these fibroblasts into two major categories: cancer-associated fibroblasts (CAFs) and normal-associated fibroblasts (NAFs) (Figure 6A; Supplementary Table S5). Among the classical markers used to denote normal fibroblasts, COL1A1 stands out prominently. At the same time, FAP is recognized as a common CAF marker that is frequently employed to discern activated fibroblasts actively shaping the tumor microenvironment. Intriguingly, we observed widespread expression of COL1A1 across all fibroblasts, whereas FAP exhibited specific and elevated expression in CAFs (Figure 6B; Supplementary Figure S6A). Furthermore, a striking prevalence of CAFs was evident in cases of primary liver cancers, while colorectal cancer metastatic to the liver cancers exhibited significantly higher levels of normal fibroblasts. We then hypothesized that CAFs may exert a distinct influence on the prognostic outcomes of liver cancer patients. Transcription factor activity analysis revealed a significant upregulation of ZNF419 in CAFs (Figure 4C), a marker previously identified in studies as indicative of immune microenvironment alterations and an adverse prognosis in cancer (Song et al., 2021).

Subsequently, we further characterised fibroblasts into subtypes based on their marker expressions (Supplementary Figure S6C). CAFs underwent division into five subtypes, while NAFs were classified into eight subtypes (Supplementary Figure S6C). Substantial variations in the prevalence of distinct fibroblast subtypes were evident across different datasets (Figure 6D; Supplementary Figure S6D). Notably, primary liver cancer significantly enriched all kinds of fibroblasts (Supplementary Figure S6E). Cellular communication analysis suggests the existence of extensive crosstalk between CAFs and CD8^+^ T cells (Supplementary Figure S7). For example, MMP2+ CAFs can regulate CD8^+^ T cells through THBS2 (Figure 6E). Existing research has implicated MMP2 as potentially upregulated in certain cancers, particularly within CAFs situated in the tumor’s periphery (Affo et al., 2017). Fibroblasts can also regulate the expression of matrix metalloproteinases such as MMP2, affecting tumour cells’ growth and metastasis (Zhou et al., 2017). Within the tumor microenvironment, CAFs wield influence over the metabolism of cancer cells, supplying them with essential metabolic substrates that, in turn, promote tumor growth. The expression of CXCL14 in CAFs is known to influence tumor growth and metastasis by recruiting immune cells and other cell types, thus facilitating their localization within tumor tissues. This process significantly impacts the tumor microenvironment, ultimately shaping the trajectory of tumor development and influencing responses to treatment. Pseudo-time analysis hinted at CAFs with high MMP2 and CXCL14 expression representing terminal subtypes that stem from normal fibroblasts (Figure 6F). This underscores the transformation of normal fibroblasts into activated cancer-associated fibroblasts during the malignant progression of cancer.

### 3.7 CAF gene signature is associated with a worse prognosis

**FIGURE 7:**
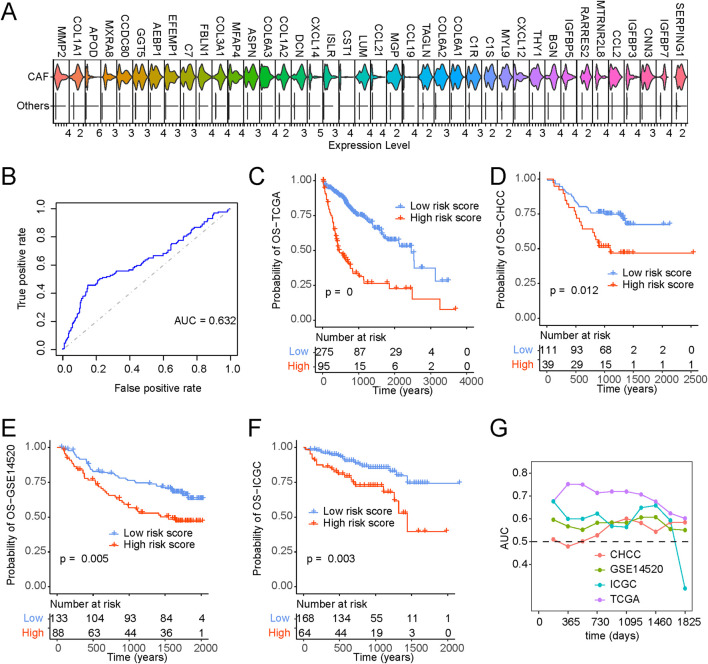
Identification of CAF-based signature and construction of risk model. **(A)** Top 40 expressed cell markers in CAFs identified by core liver single-cell datasets. **(B)** ROC curve of risk scores and OS status. **(C–F)** Kaplan-Meyer plot of patients with high and low-risk scores of **(C)** TCGA, **(D)** CHCC, **(E)** GSE14520, **(F)** ICGC, patients with liver cancer as determined by risk model. **(G)** Time-dependent AUC value in TCGA, CHCC, GSE14520 and ICGC.

We procured single-cell gene expression profiles, followed by identifying genes significantly upregulated within CAFs compared to other cell types. From this analysis, we selected the top 40 differentially expressed genes for further investigation (Figure 7A). To pinpoint optimal prognostic gene biomarkers from this set of 40 CAF-specific genes, we employed a LASSO-Cox regression model on the gene expression profiles and clinical data derived from TCGA LIHC samples (Supplementary Table S6). This approach led to the formulation of a nine-gene signature model, where the risk score computation was structured as follows: risk score = 0.091*THY1 + 0.199*CNN3 + 0.089*IGFBP3-0.066*IGFBP7-0.031*SERPING1-0.066*C7-0.017*RARRES2-0.096*C1S-0.015*CXCL14. A detailed exposition of the model parameters can be found in Supplementary Figure S8A. Notably, the predicted risk score exhibited pronounced distinctions across various survival statuses (Figure 7B; Supplementary Figure S8B).

Furthermore, Kaplan-Meier survival analysis and Cox regression substantiated that patients bearing higher risk scores faced significantly bleaker prognoses (Figure 7C). Figures 7D–F provide a comprehensive portrayal of the risk score’s robust performance across diverse datasets, affirming its consistency in prognosticating survival probabilities. In addition, our exploration encompassed time-dependent ROC curve analysis, further underscoring the substantial potential of the risk score in forecasting patient survival statuses (Figure 7G). To enhance the clinical utility of the risk score, we developed a nomogram capable of predicting 1-year, 3-year, and 5-year overall survival probabilities. As illustrated in Supplementary Figure S9A, the nomogram analysis demonstrated minimal deviation between the risk score and actual overall survival probabilities at these designated time points (Supplementary Figures S9A–D).

## 4 Discussion

This study presents a comprehensive analysis of liver cancer using single-cell sequencing data, establishing a detailed single-cell atlas and uncovering crucial insights into the tumor microenvironment, immune phenotypes, genetic mutations, and their impact on patient prognosis. The study identifies distinct immune phenotypes, reveals the influence of genetic mutations on immune cell composition, and highlights the significance of fibroblasts, particularly cancer-associated fibroblasts, in patient survival. A CAF gene signature model is developed as a prognostic tool, offering potential clinical applications.

Our analysis of immune cell composition revealed distinct immune phenotypes within liver cancer patients. These phenotypes, including immune-deserted, T cell-infiltrated, B cell-infiltrated, and macrophage-infiltrated types demonstrated variations in enriched cell types. D-type tumors exhibit a profound immunosuppressive microenvironment facilitated by VTN and MDK. These ligands, through interactions with integrin receptors such as ITGB1 and ITGA8, reduce T cell activation and migration while promoting immune tolerance. This mechanism establishes an exclusionary barrier that impedes immune cell infiltration. Conversely, in T-type tumors, NAMPT is a critical modulator of T cell metabolism and immune responses. By maintaining NAD + levels, NAMPT supports T cell proliferation and enhances the differentiation and activity of Th1 cells, fostering an environment conducive to strong anti-tumor immunity. Further investigation into tumor cell gene expression profiles across these subtypes highlighted significant differences in fatty acid metabolism, inflammation, and Toll-like receptor signaling pathways. The identification of an immune-deserted phenotype in liver cancer aligns with recent research emphasizing the role of immune evasion in tumor progression (Siddiqui and Glauben, 2022; Li et al., 2005). Recent studies have investigated immunotherapeutic strategies, including immune checkpoint inhibitors, to reverse such immunosuppression in liver cancer (Cheng et al., 2020). The upregulation of fatty acid metabolism in immune-deserted tumors underscores the significance of metabolic reprogramming in immune evasion. Recent publications have elucidated the interplay between tumor cell metabolism and immune cell function (Leone and Powell, 2020), highlighting metabolic pathways as potential therapeutic targets.

Moreover, we investigated the immune cell composition differences between non-metastatic colorectal cancer, primary liver cancer, and colorectal cancer metastatic to the liver. These analyses indicate the critical involvement of mast cells in colorectal cancer metastasis to the liver and suggest potential therapeutic strategies. Mast cell-related metabolic pathways, including inositol phosphate metabolism and glycosaminoglycan biosynthesis, could be targeted to mitigate metastasis. Existing therapies such as tyrosine kinase inhibitors (e.g., imatinib) (Pardanani et al., 2003), which suppress c-KIT-mediated mast cell activation, have been used to treat systemic mastocytosis. While not specifically studied in CRC liver metastasis, targeting mast cells through c-KIT inhibition could be explored, given the role of mast cells in promoting metastasis through immunosuppressive and pro-angiogenic activities. Additionally, LiCl (lithium chloride) (Wei et al., 2012), an inhibitor of inositol phosphate metabolism, and heparin analogs, which block glycosaminoglycan biosynthesis, represent promising avenues for therapeutic intervention. Other approved drugs, such as masitinib (Ettcheto et al., 2021) (a selective tyrosine kinase inhibitor) and omalizumab (Serrano‐Candelas et al., 2016) (an anti-IgE antibody), could also be evaluated for their potential to inhibit mast cell activity and prevent metastasis. These strategies underscore the translational relevance of targeting mast cells and their metabolic pathways in colorectal cancer metastasis. Integrating genetic profiles and single-cell data allowed us to identify significant associations between genetic mutations, immune cell composition, and patient survival. TP53 mutations were correlated with mast cell and T cell infiltration, while CTNNB1 mutations exhibited distinct associations with endothelial cells and fibroblasts. These findings shed light on the interplay between genetic alterations and the immune microenvironment in liver cancer.

Furthermore, Our analysis revealed that fibroblast components, particularly CAFs, were strongly associated with poor prognosis in liver cancer. CAFs contribute to tumor progression through multiple mechanisms, including immune suppression, angiogenesis, and extracellular matrix remodeling. Key signaling pathways, such as TGF-β signaling (Busch et al., 2015) and fibroblast growth factor (FGF) signaling (Cristinziano et al., 2021), mediate tumor-CAF interactions, promoting cancer cell proliferation and invasion. For instance, CAFs expressing MMP2 interact with tumor cells via THBS2, enhancing extracellular matrix degradation and tumor cell motility (Drev et al., 2017). Similarly, CXCL14, highly expressed in CAFs, recruits immune and stromal cells to support tumor growth (Westrich et al., 2020). The distinct prevalence of CAFs in primary liver cancers and NAFs in colorectal cancer liver metastases suggests a dynamic role for fibroblasts at different stages of cancer progression. In primary liver cancers, CAFs actively shape a pro-tumorigenic microenvironment through pathways such as TGF-β, FGF signaling, and integrin-mediated adhesion, promoting tumor cell invasion and immune evasion. In metastatic lesions, the relative abundance of NAFs may indicate a transitional microenvironment, potentially reflecting a reduced ability to support metastatic growth compared to primary tumors. This differential fibroblast composition highlights the need to explore CAF-targeted therapies, such as inhibitors of TGF-β signaling or matrix metalloproteinases, to improve patient outcomes in liver cancer.

This study relies on publicly available single-cell sequencing datasets, which may have inherent biases and variations in data quality. Future studies should consider incorporating additional datasets and validating findings in independent cohorts. While the study provides detailed cell annotations based on marker genes, the accuracy of these annotations may be influenced by the choice of markers and the potential presence of rare cell types. A more in-depth validation of cell types is warranted. Besides, although the study identifies potential interactions and pathways, functional experiments are needed to validate the biological significance of these findings. *In vitro* and *in vivo* experiments would better understand the mechanisms involved. The prognostic gene signature model requires further validation in clinical settings.

## 5 Conclusion

Our study provides comprehensive insight into the immune phenotypes, genetic associations, and tumor microenvironment interactions in liver cancer. The identification of key genes, pathways, and prognostic markers offers valuable information for understanding liver cancer progression and may contribute to the development of targeted therapies and personalized treatment strategies.

## Data Availability

The original contributions presented in the study are included in the article/Supplementary Material, further inquiries can be directed to the corresponding authors.

## Abbreviations

CAFs, Cancer-associated fibroblasts; TCGA, The Cancer Genome Atlas; HBV, Hepatitis B Virus; scCRLM, Single-Cell Colorectal Cancer Liver Metastases; ORs, odds ratios; KEGG, Kyoto Encyclopedia of Genes and Genomes; GSEA, Gene Set Enrichment Analysis; ssGSEA, single-sample Gene Set Enrichment Analysis; ROC, receiver operating characteristic; LM, metastatic lesions of livers; PL, primary liver; PC, primary colorectal cancers; CRC, non-metastatic colorectal cancers; CNV, copy number variation; TLR, Toll-like receptor; NAFs, normal-associated fibroblasts.
