# Supplementary material for: Deciphering the heterogeneity and plasticity of the tumor microenvironment in liver cancer provides insights for prognosis
Source: Front Pharmacol. 2025 Jan 30;16:1495280. doi: 10.3389/fphar.2025.1495280 (PMC11821625; doi:10.3389/fphar.2025.1495280)
Supplement: Supplementary file 4 [file DataSheet1.pdf]

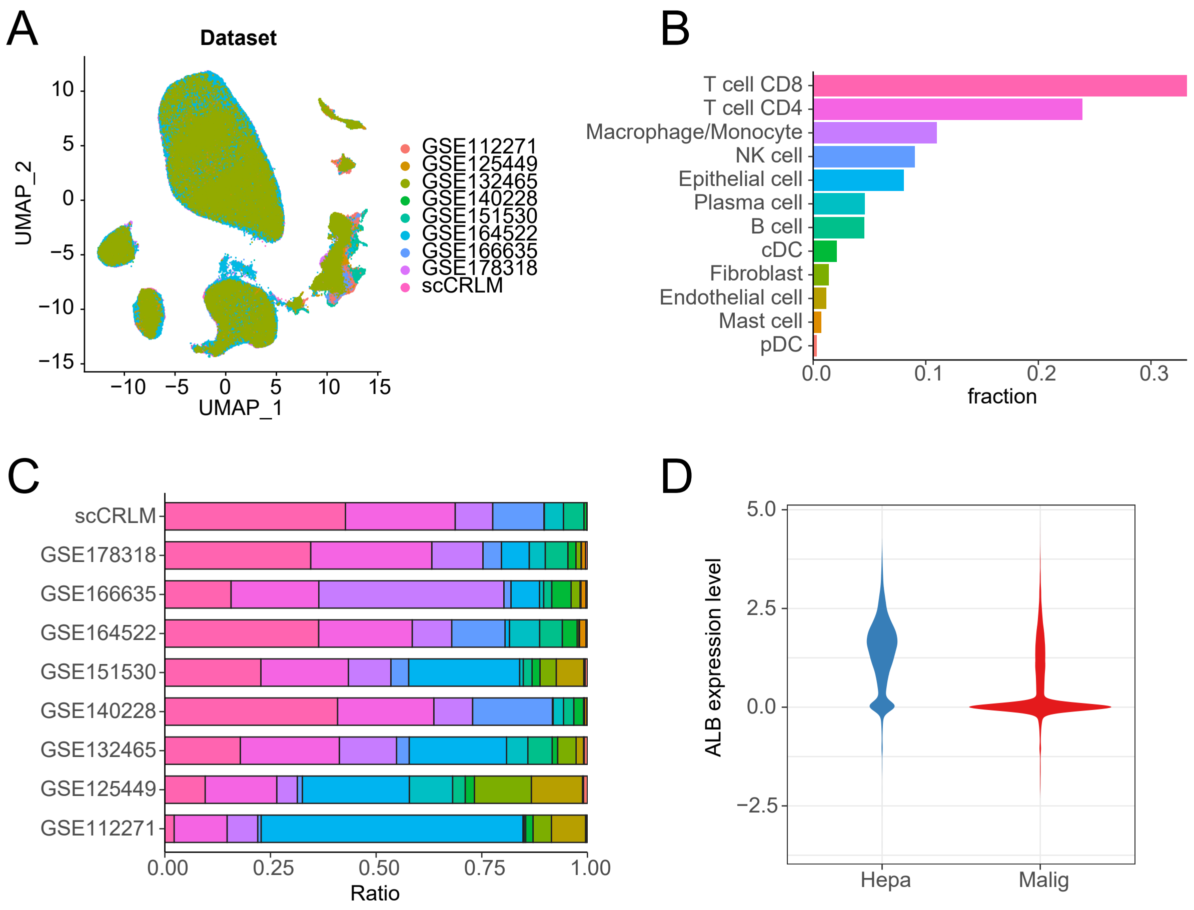

Figure S1. Composition of the liver cancer single-cell atlas. (A) UMAP of core liver cancer atlas from different datasets. (B) Cell type fractions in the core atlas. (C) Fractions of cell types and sample origins per study. (D) Expression levels of liver marker gene ALB between normal epithelial cells and malignant cells.

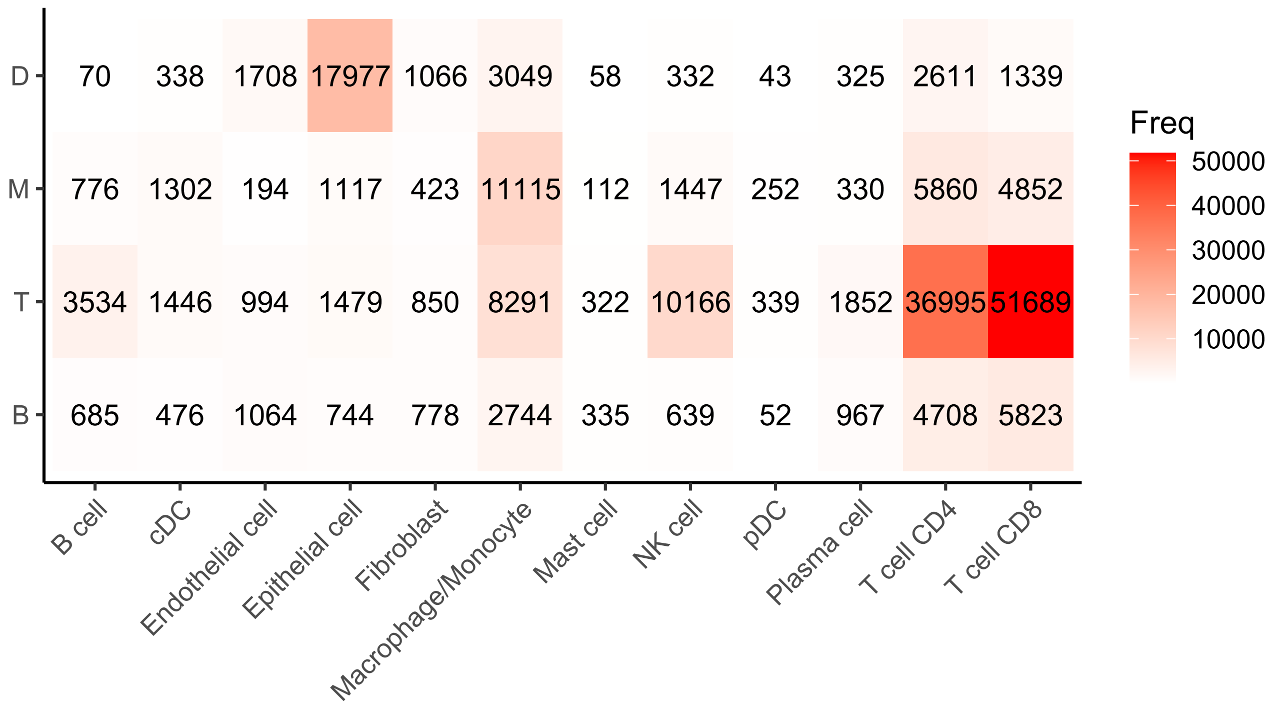

Figure S2. Tumor microenvironment subclusters by the contributing datasets. Left column shows the tumor microenvironment subclusters, the heatmap depicts the number of cells per subclusters.

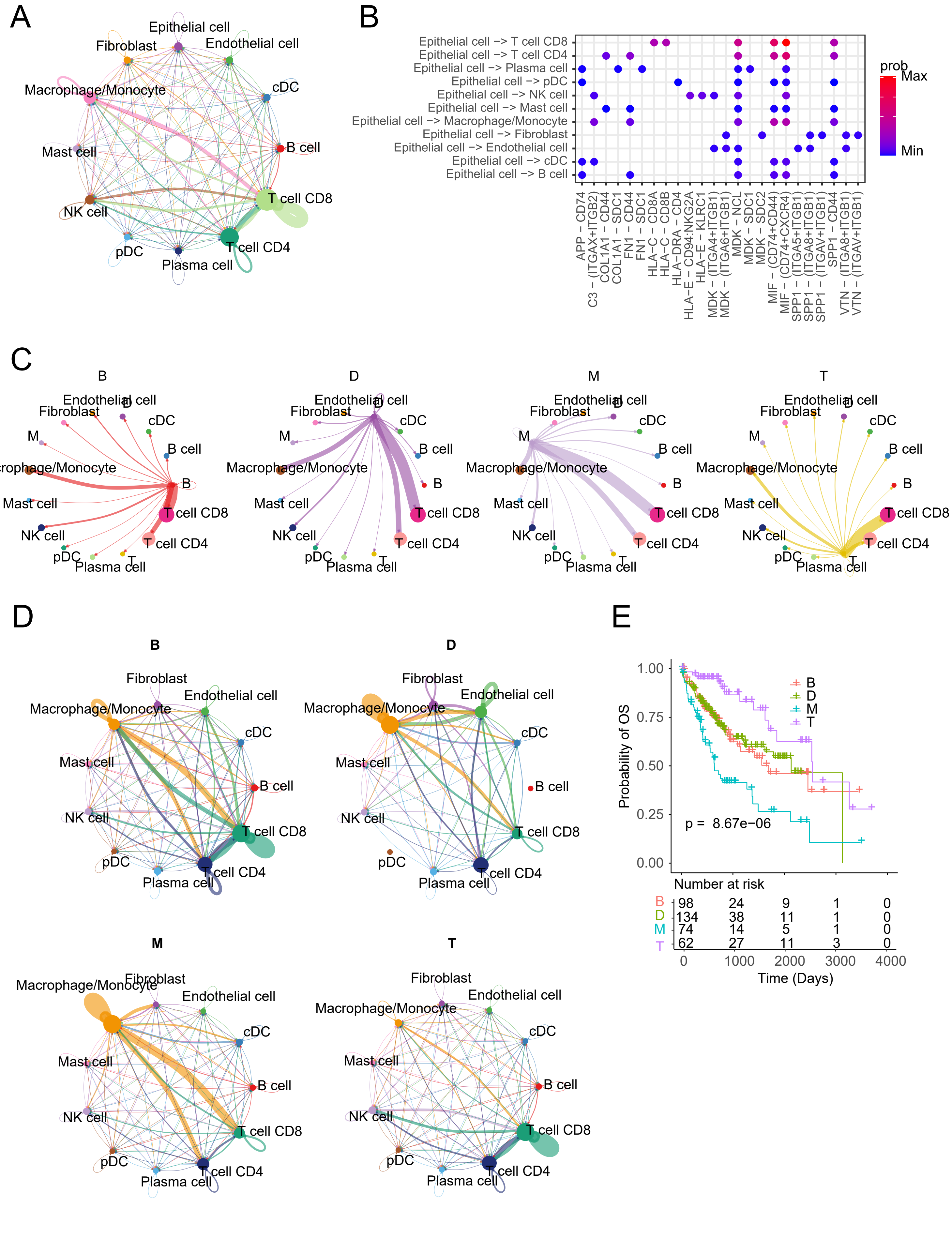

Figure S3. Crosstalk between cancer cells and immune cells. (A) Circos plot of the cellular crosstalk of all cells. (B) Cancer-immune cell crosstalk. (C) Circos plot of the cellular crosstalk of cancer cells toward the major immune cells in each patient subcluster. (D) Circos plot of the cellular crosstalk between different TME cells in each patient subcluster. (E) Kaplan-Meyer plot of patients across different TME groups of TCGA patients with liver cancer. P-value has been determined using CoxPH regression using tumor stage and age as covariates.

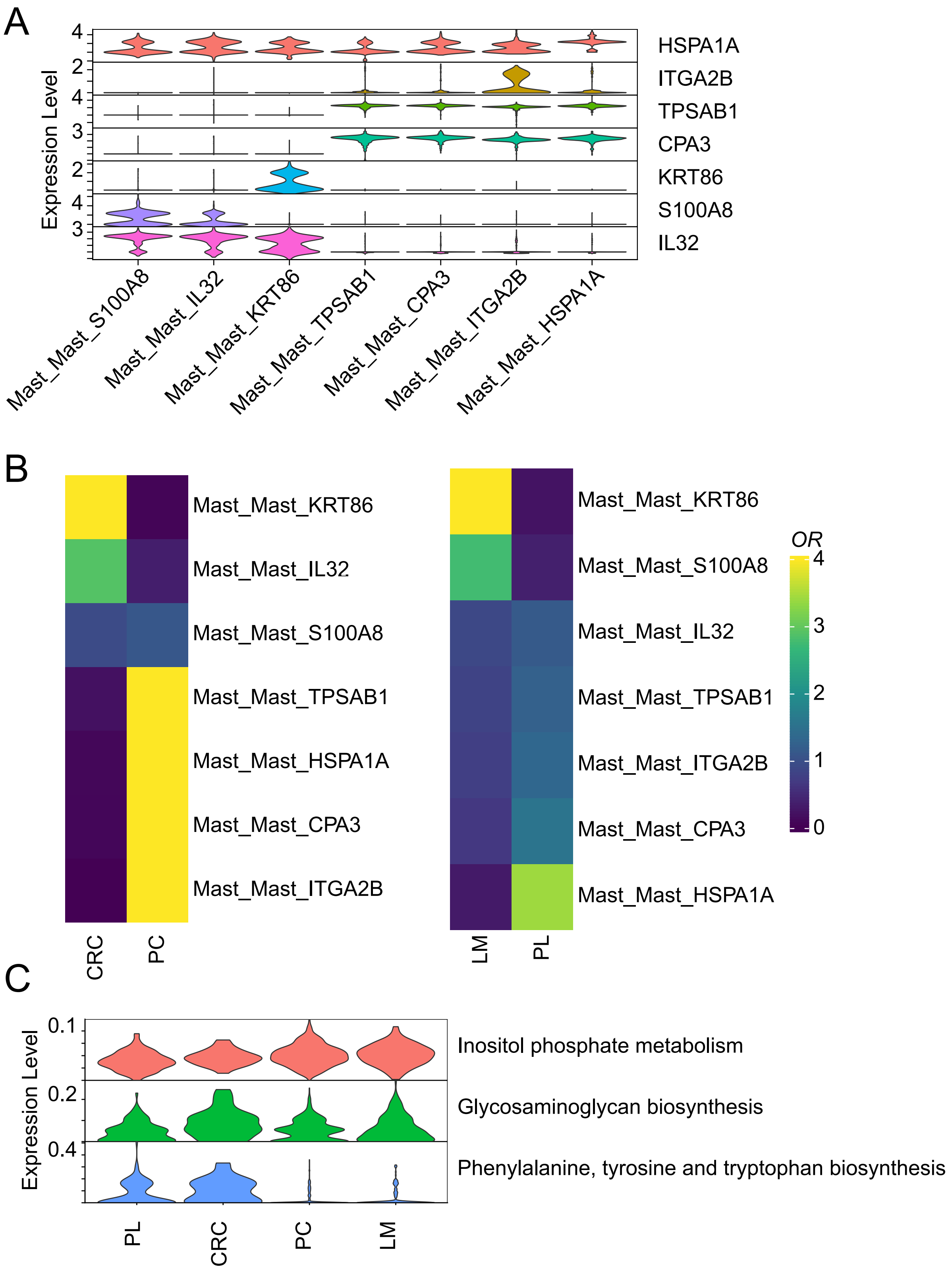

Figure S4. Association of cellular composition and distinct genotypes and survival in the TCGA data. (A) UMAP of cell type marker genes used for cell-type annotation. (B) Cell-type composition differences of mast cells between different tumor origins. (C) Differential activation of metabolic pathways in mast cells between different tumor origins.

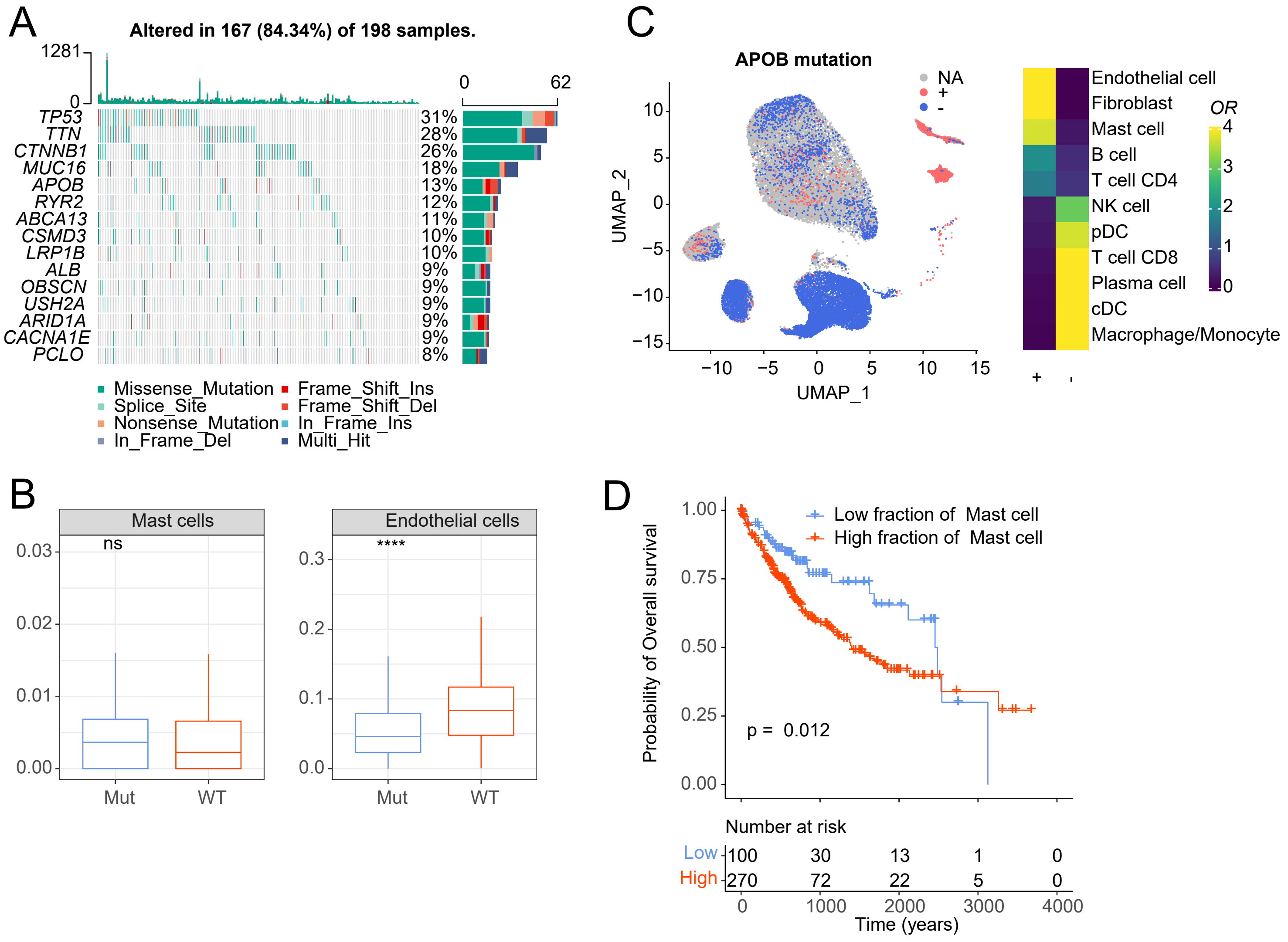

Figure S5. Association of cellular composition and distinct genotypes and survival in the TCGA data. (A) Mutation landscape of TCGA liver cancer samples. (B) Cell type fractions difference between TP53 mutation and wild-type samples. (C) Association of cellular composition with APOB mutation in patients with LIHC. (D) Kaplan-Meyer plot of patients with high and low mast cell fractions of TCGA patients with liver cancer as determined by deconvolution with xCell. P-value has been determined using CoxPH regression using tumor stage and age as covariates

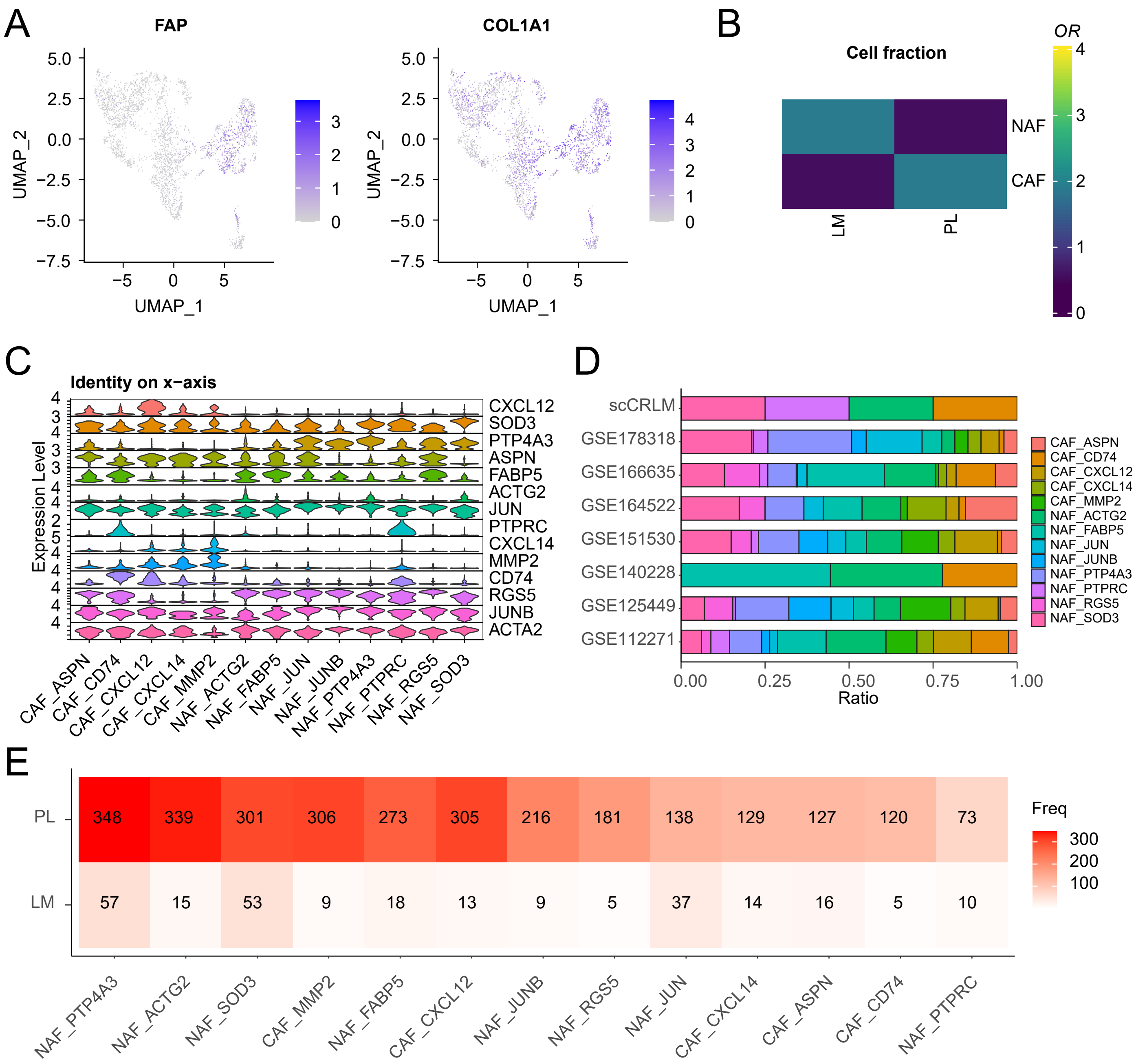

Figure S6. Tissue-resident fibroblasts subtypes in liver cancer. (A) UMAP of cell type marker genes used for cell-type annotation. (B) Fibroblasts cell-type composition between different tumor tissues. (C) UMAP of cell type marker genes used for cell-type annotation. (D) Fibroblasts cell type fractions in the core atlas. (E) Fibroblasts subclusters composition among different tumor tissues

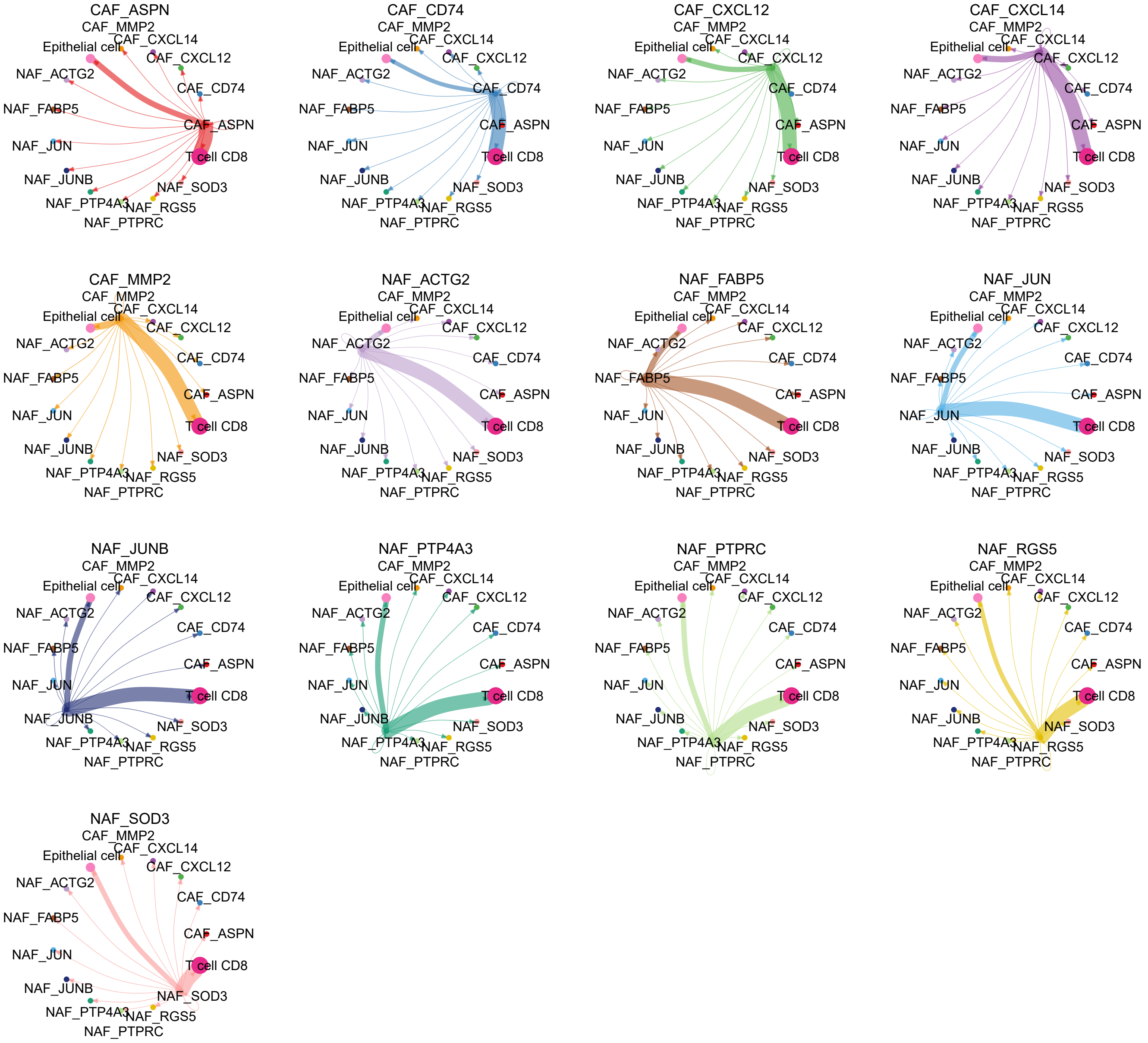

Figure S7. Circos plot of the cellular crosstalk of fibroblast subclusters toward other cells.

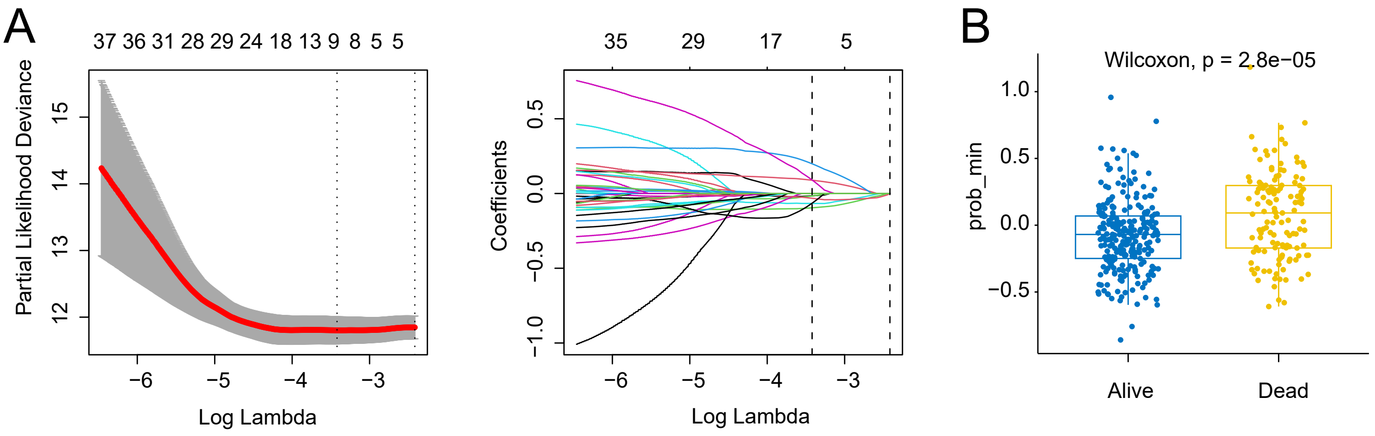

Figure S8. Marker gene identification and model construction. (A) The partial likelihood deviance and regression coefficients were calculated by multivariate Cox regression. (B) Boxplot shows the risk score difference between samples with different OS statuses.

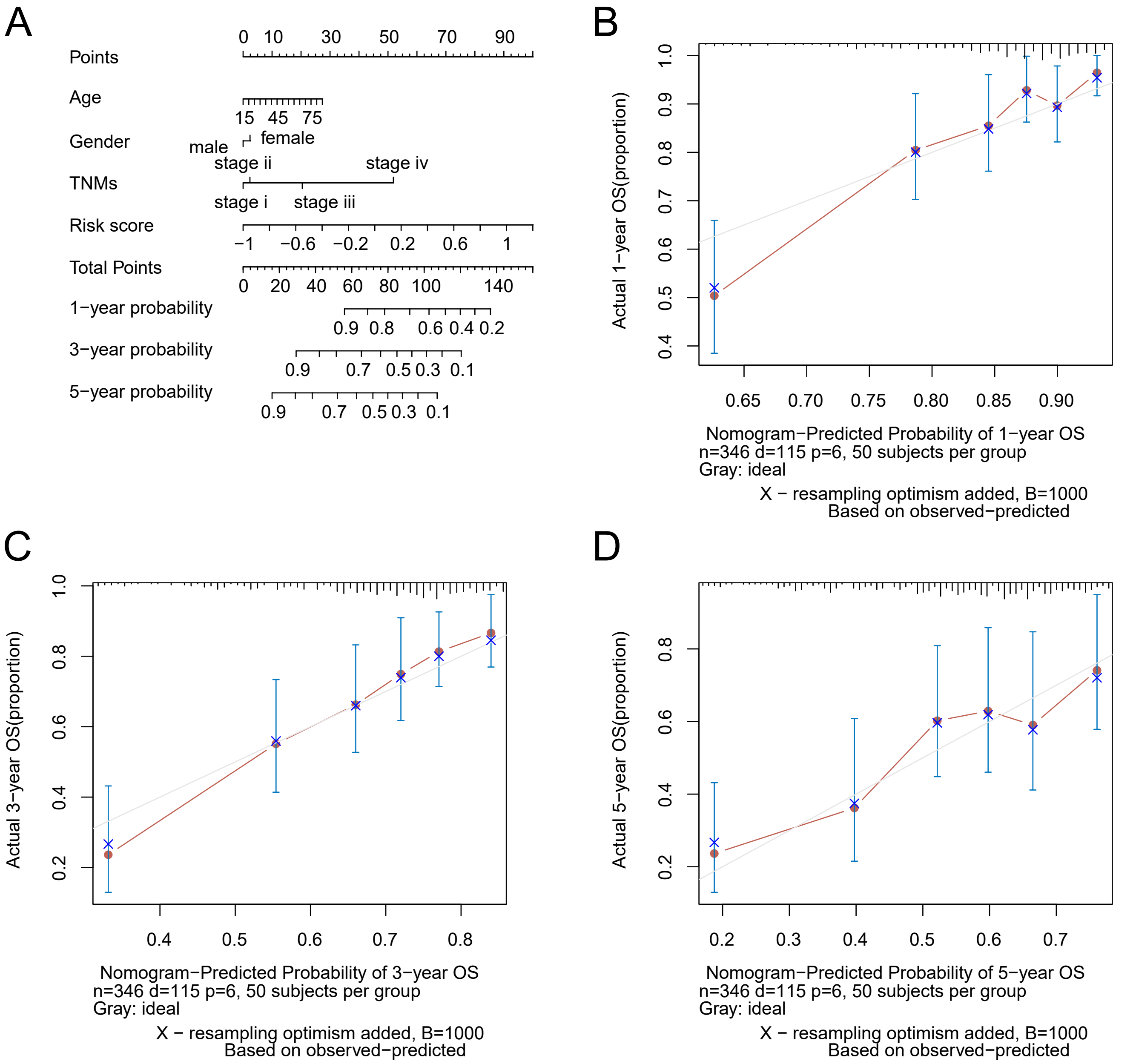

Figure S9. Nomogram analysis. (A) Nomogram composed of age, gender, TNM stage and risk score for the prediction of 1-, 3-, and 5-years OS probability. Calibration plot for the evaluation of the nomogram in predicting 1-year (B), 3-years (C), and 5-years (D) OS probability.
